# A Case Report of Scarless Direct Access to the Infraorbital Rim Using a Retroseptal Transconjuctival Approach

**DOI:** 10.7759/cureus.3836

**Published:** 2019-01-07

**Authors:** Kalarikkal Mukundan Harish, James Antony Bhagat, Guruprasad Tulasidas

**Affiliations:** 1 Oral and Maxillofacial Surgery, Ragas Dental College and Hospital, Chennai, IND; 2 Oral and Maxillofacial Surgery, Adhiparasakthi Dental College, Chennai, IND; 3 Oral and Maxillofacial Surgery, SRM Dental College, Chennai, IND

**Keywords:** retroseptal transconjuctival approach, scarless approach, direct access technique

## Abstract

A variety of approaches have been documented in the literature for accessing the infraorbital rim and orbital floor in cases of fractures involving orbitozygomatic maxillary complex fractures. Various transcutaneous approaches like infraorbital, subtarsal, and subciliary approaches have been employed traditionally to access these regions. However, significant postoperative complications are associated with these approaches. The transconjunctival approach to access the infraorbital rim and orbital floor has recently been re-evaluated. We present a case of a patient with a zygomaticomaxillary complex fracture in which the infraorbital rim was fixed using a transconjunctival retroseptal approach.

## Introduction

The incidence of oral and maxillofacial injuries is constantly on the rise and may be attributed to the surge in the number of motor vehicles and noncompliance of riders in wearing helmets or seat belts. The zygoma, being the most prominent bone on the face, is commonly affected in maxillofacial injuries and is frequently involved in combined orbitozygomatic maxillary complex fractures [1]. The various transcutaneous approaches to the infraorbital rim and orbital floor include subciliary, subtarsal, and infraorbital approaches, and they have been associated with a significant number of complications like ectropion, scleral show, and eyelid retraction [1]. In 1924, Bourguet initially used a transconjunctival approach for lower eyelid blepharoplasty [2]. The same technique was employed later by Tenzel and Miller to access small orbital floor fractures [2]. It was further used by Tessier to access the orbit in patients with craniofacial dysostoses [3]. However, with the progressive use of transconjunctival incisions, the rate of complications was drastically reduced. Also, the absence of a visible scar is a great advantage in executing a transconjunctival incision. The retroseptal transconjunctival incision has an additional advantage in offering direct access to the inferior orbital rim and floor without violating the orbital septum. We present a case of zygomatic maxillary complex fracture treated using a retroseptal transconjunctival approach.

## Case presentation

**Figure 1:**
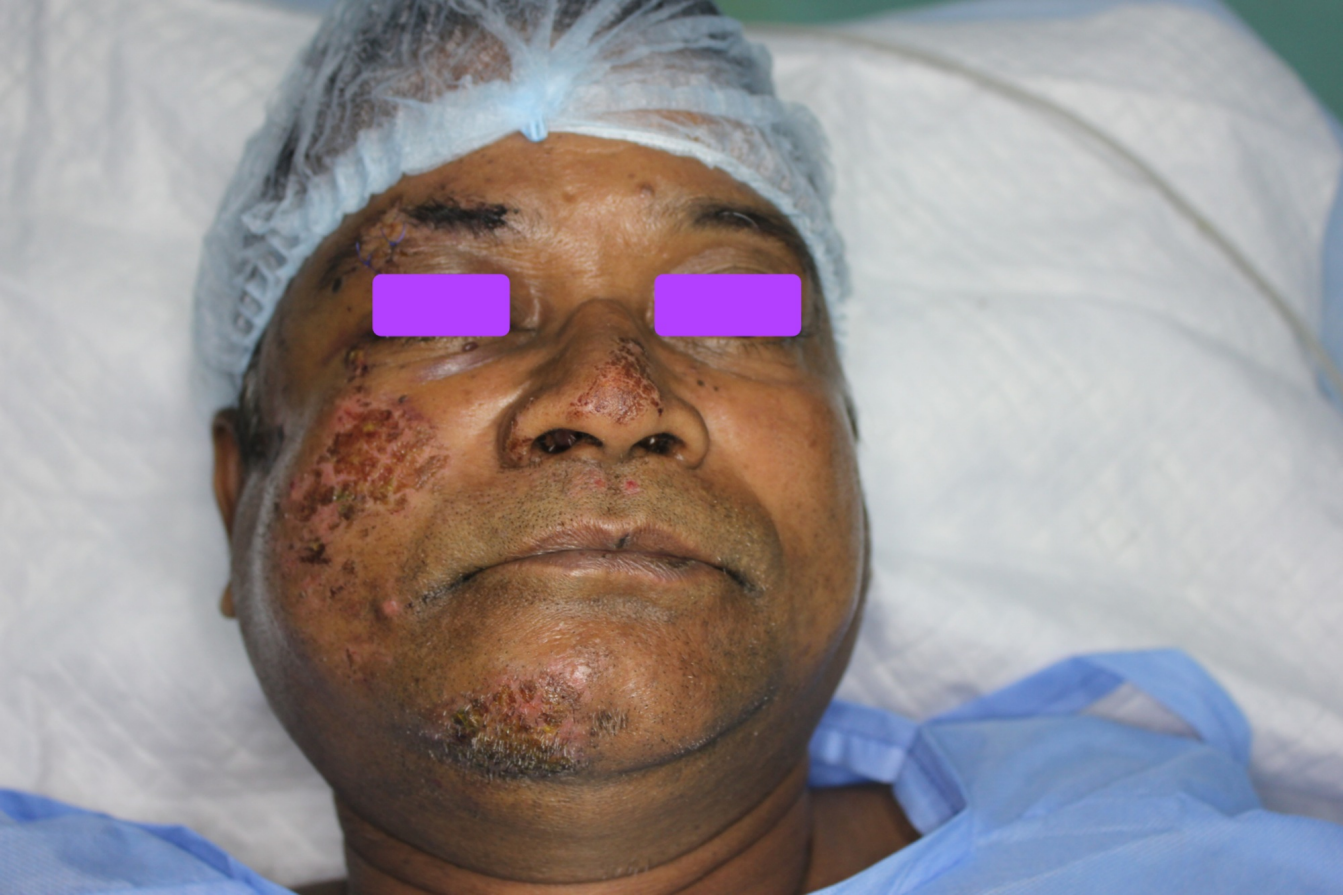
Frontal clinical picture exhibiting abrasions over the right zygoma and chin region

A 57-year-old male patient reported to our private practice with injuries to his cheekbone attributed to a road traffic accident. He reported sustaining a fall from a two-wheeler (motorcycle) 24 hours prior to presentation, resulting in an impact to his face. He was not wearing a helmet at the time of impact. He was stable at the time of presentation, and he had no known history of loss of consciousness, vomiting, or amnesia (Figure 1). He was referred to a general physician and a neurosurgeon for further examination and to obtain clearance to proceeding with surgical management of the fractured facial bones. The patient was again referred to us once deemed fit to undergo surgical management of facial bone fracture under general anesthesia. A detailed ophthalmic examination revealed no visual disturbances. The patient reported he has diabetes managed via medication for the past seven years.

**Figure 2:**
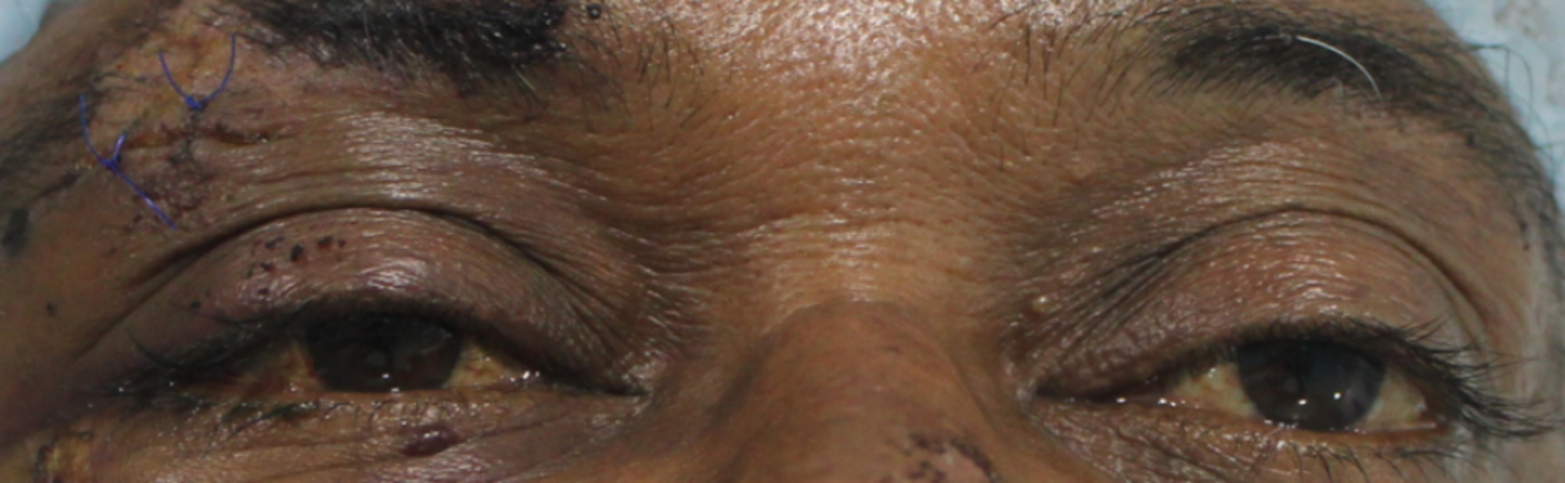
Close-up image of eyes demonstrating the lack of an obvious subconjunctival hemorrhage

On extra oral examination, we noted swelling and abrasion over the right zygomatic region. We found no obvious subconjunctival hemorrhage, diplopia, or enophthalmos (Figure 2). The patient exhibited normal ocular movements in all gazes. He had no other lacerations or soft tissue injury on his face. An intraoral examination revealed a normal occlusion; we saw no signs of fracture or mobility of any teeth. The patient had a mouth opening of 36 mm. The temporomandibular joint movements were normal, and there was no restriction or difficulty in opening his mouth. We noted tenderness in the right zygomaticomaxillary buttress and the right infraorbital rim. An infraorbital step was noted on the right side. We found no evidence of sublingual hematoma. Crepitus was noted in the right zygomaticomaxillary buttress region.

**Figure 3:**
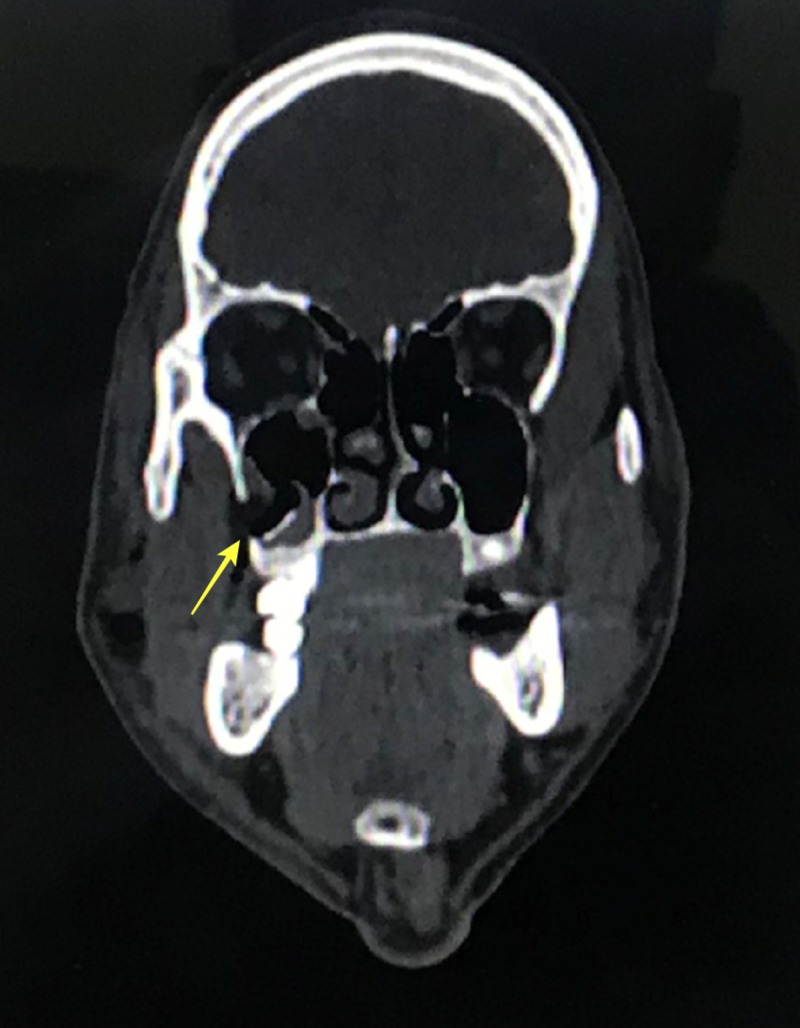
Coronal computed tomography view demonstrating fracture in the right zygomatic maxillary buttress region

**Figure 4:**
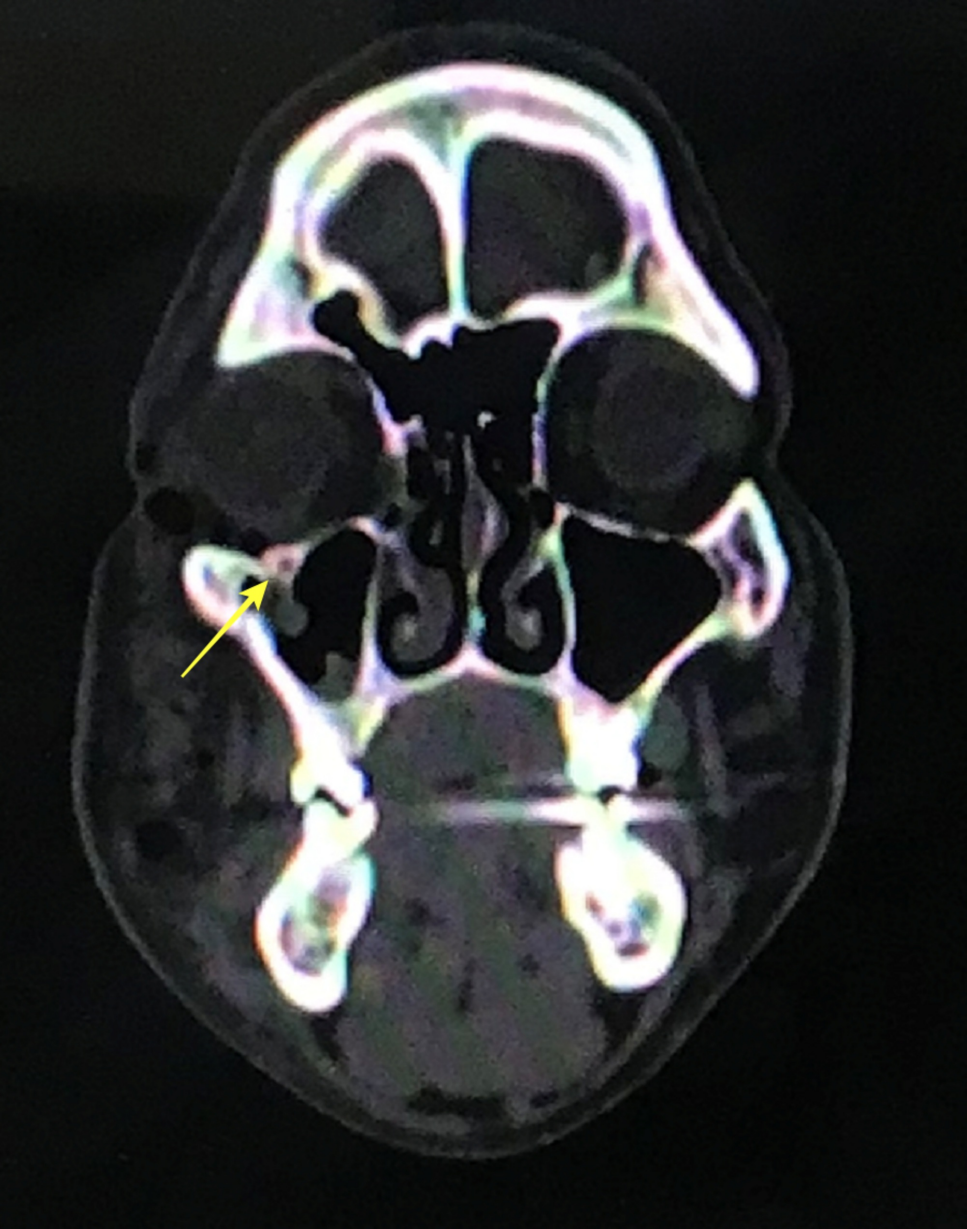
Coronal computed tomography view demonstrating fracture at the infraorbital rim

A computed tomography (CT) scan revealed a fracture of his right zygomaticomaxillary buttress region and right infraorbital rim region (Figures 3-4).

**Figure 5:**
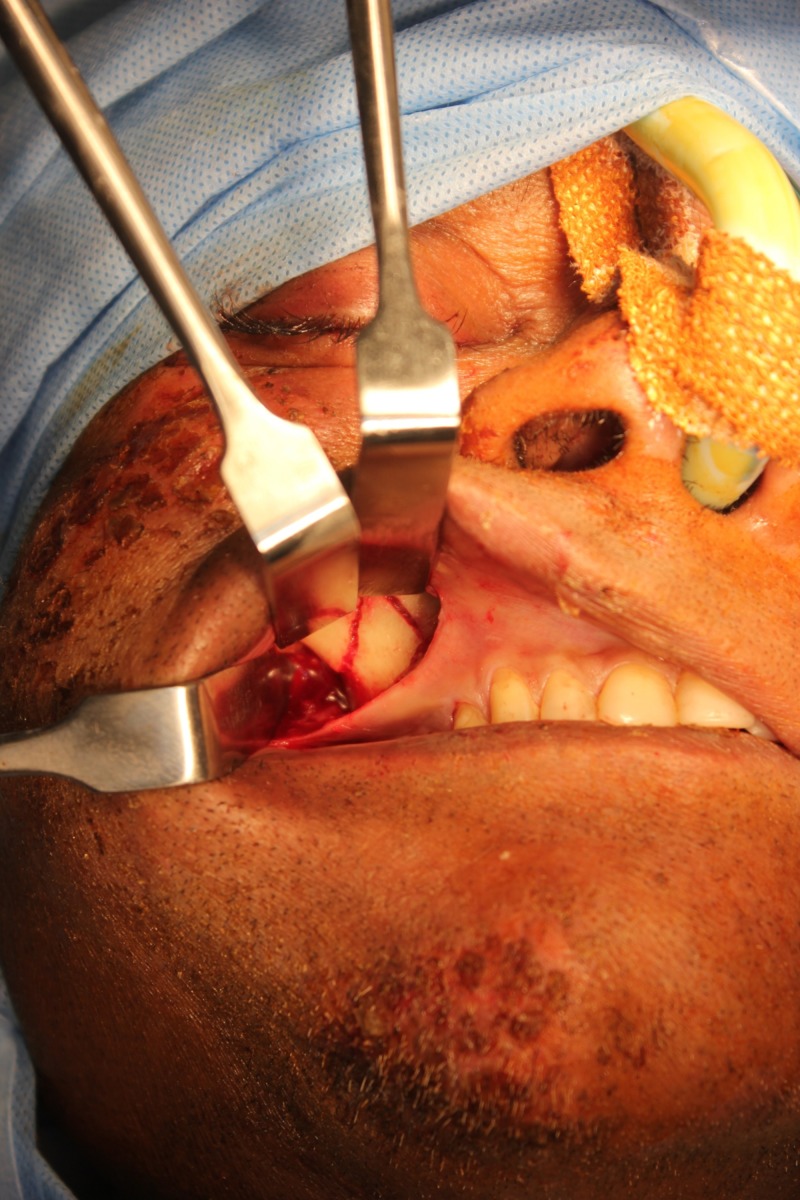
Surgical exposure of the fracture at the right zygomatic maxillary buttress region

**Figure 6:**
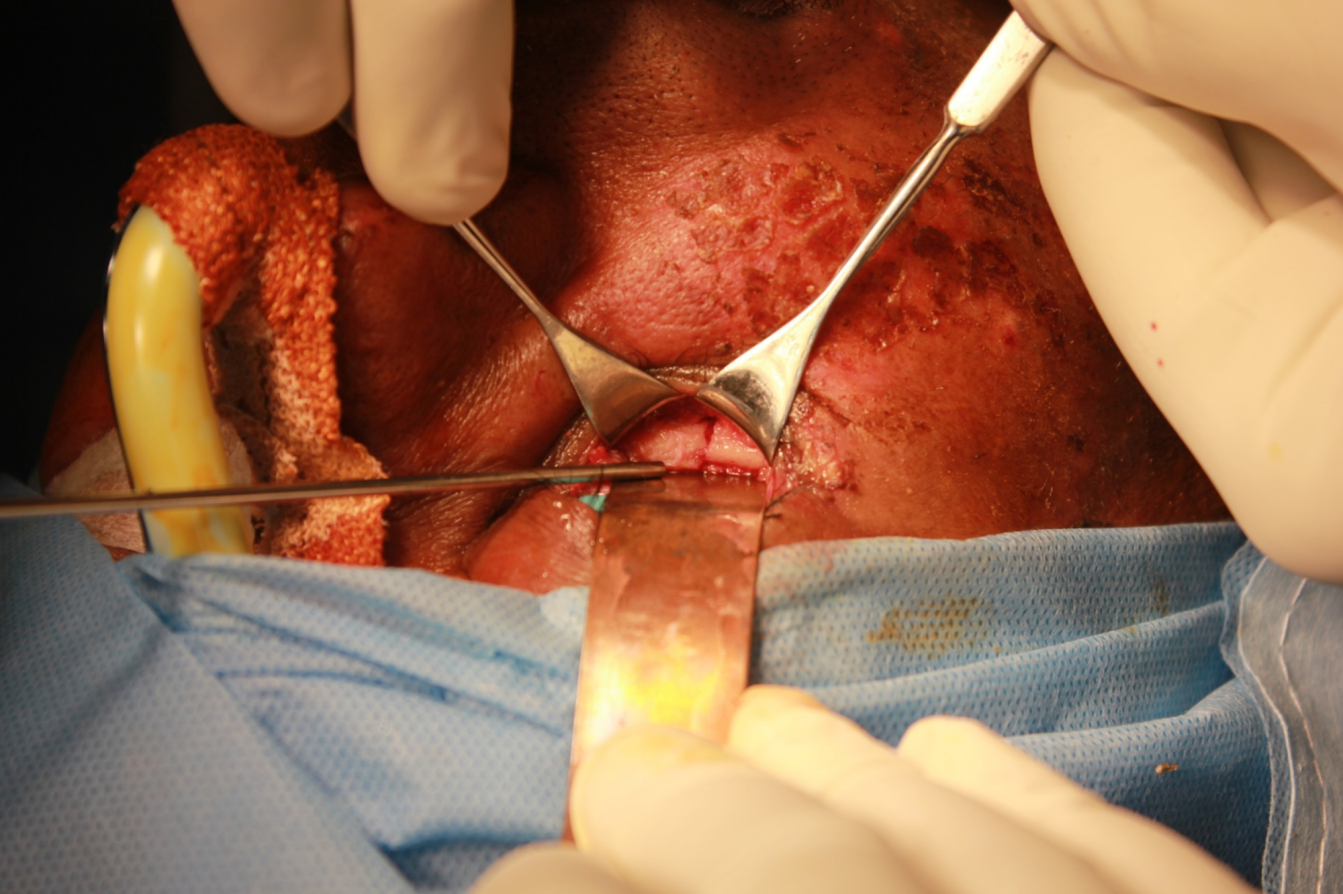
Surgical exposure (head end view) of the fracture at the infraorbital rim using a retroseptal transconjunctival approach

**Figure 7:**
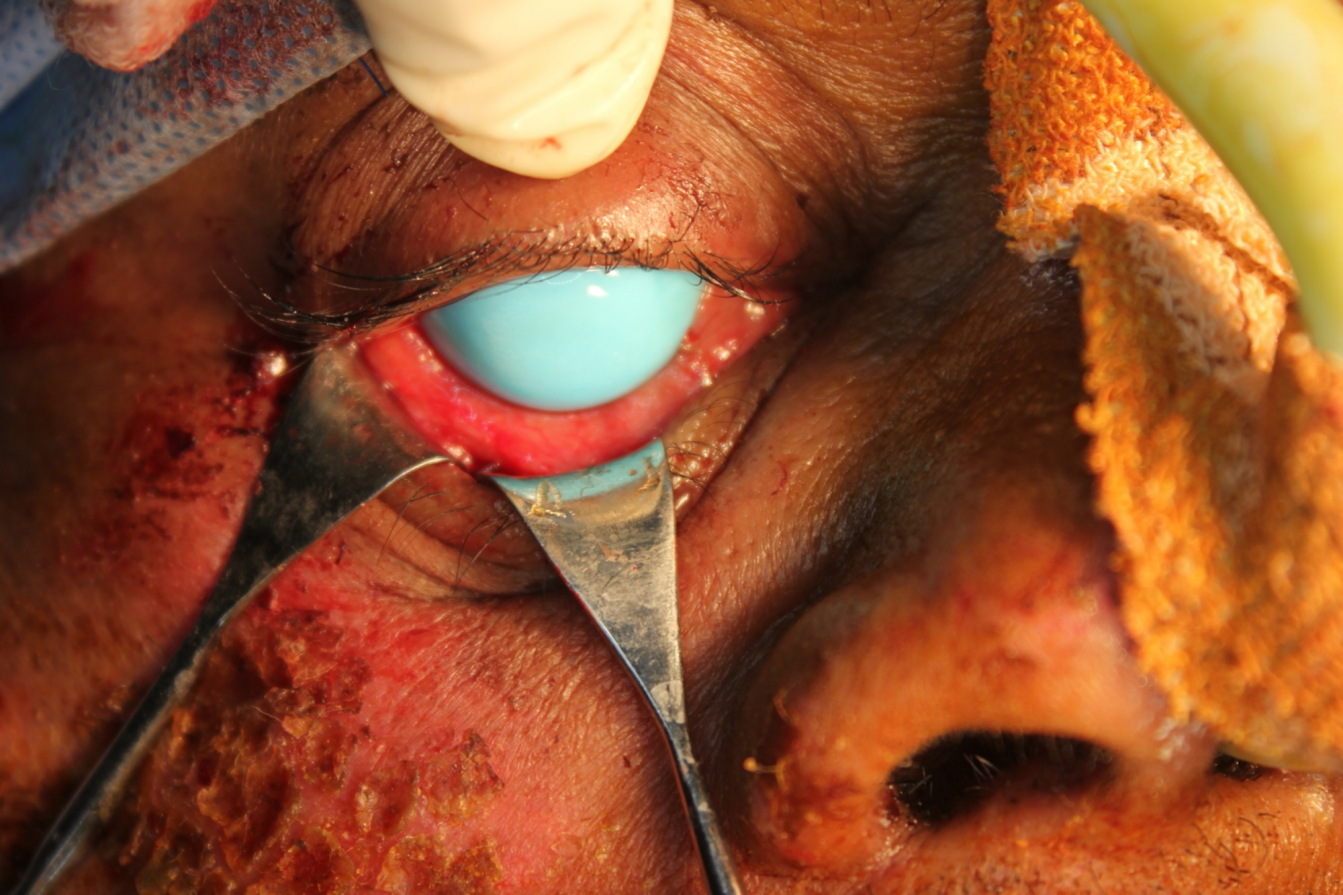
Placement of the corneal shield to prevent injuries to the cornea and the eyeball

**Figure 8:**
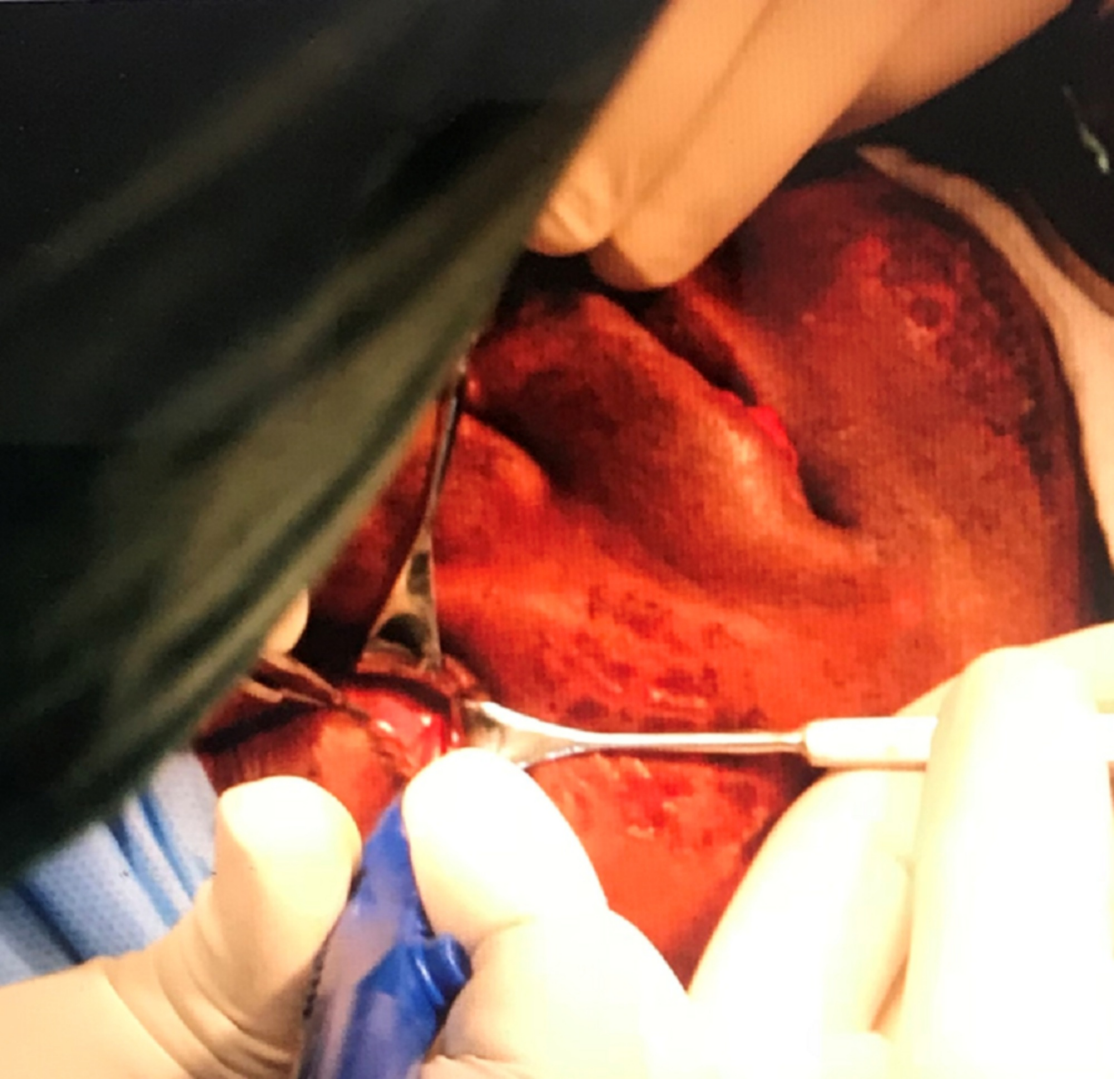
Placement of a retroseptal transconjunctival incision using Colorado tip electrocautery

**Figure 9:**
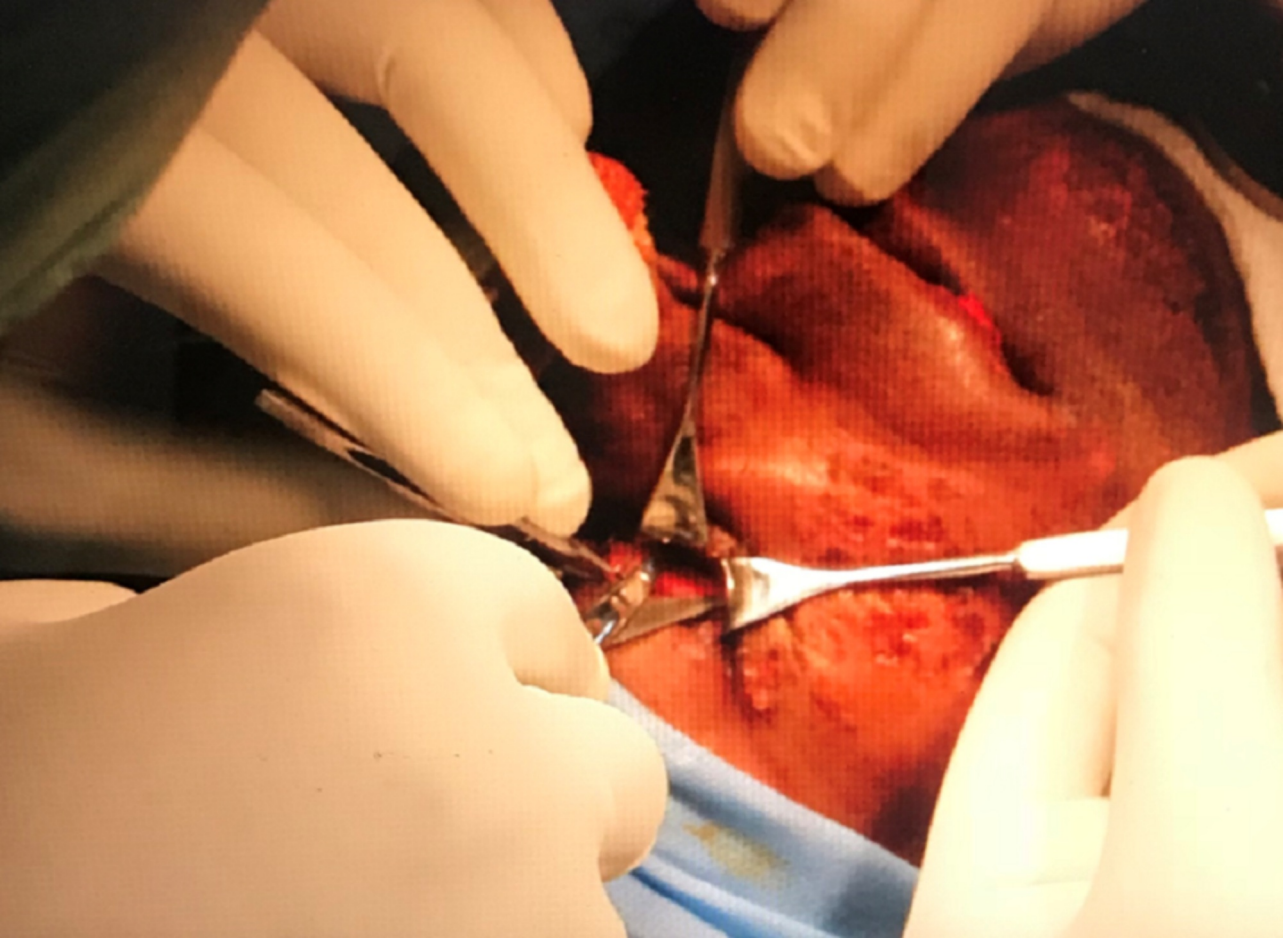
Dissection performed to expose the infraorbital rim

**Figure 10:**
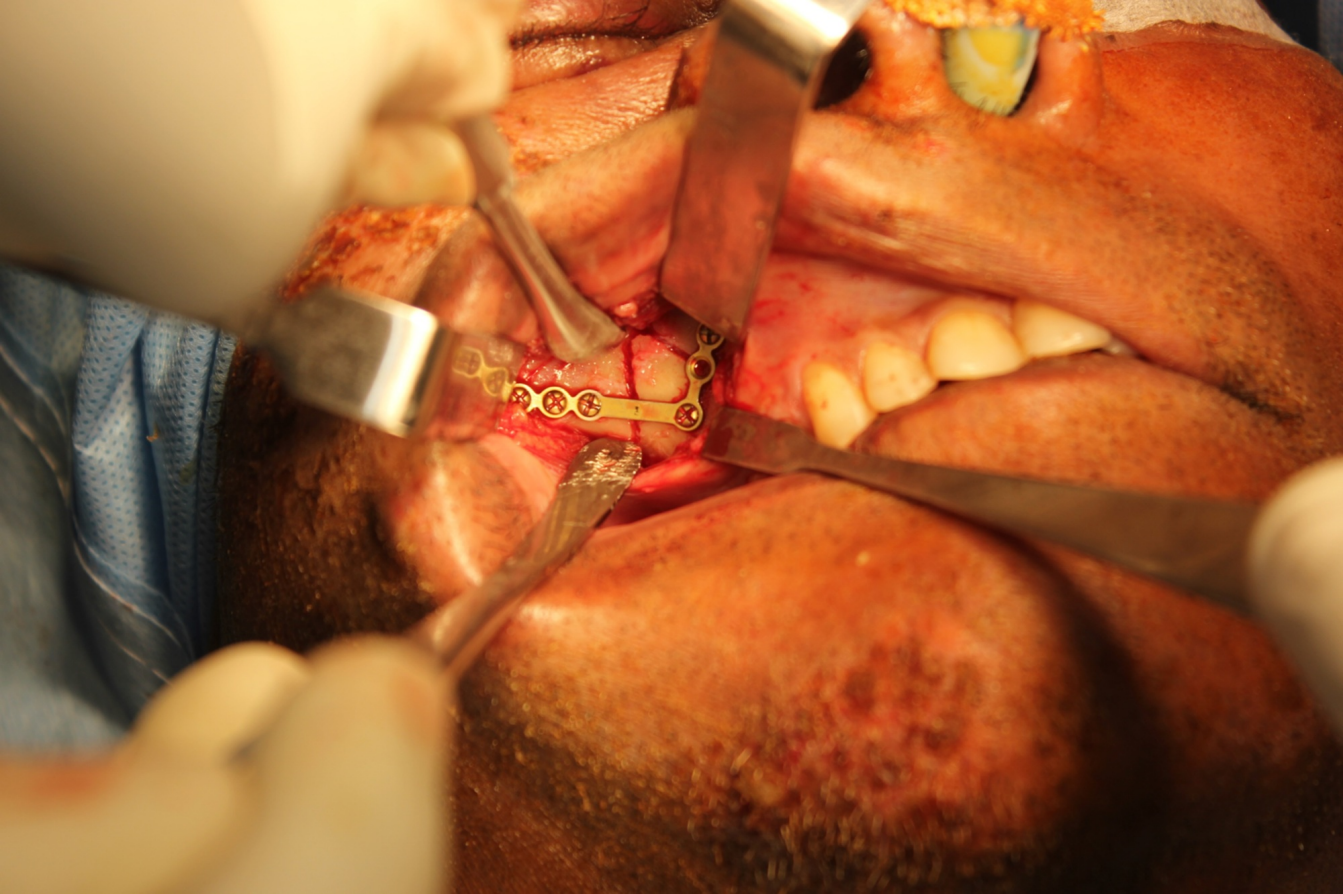
Miniplate fixation of right zygomatic maxillary buttress

**Figure 11:**
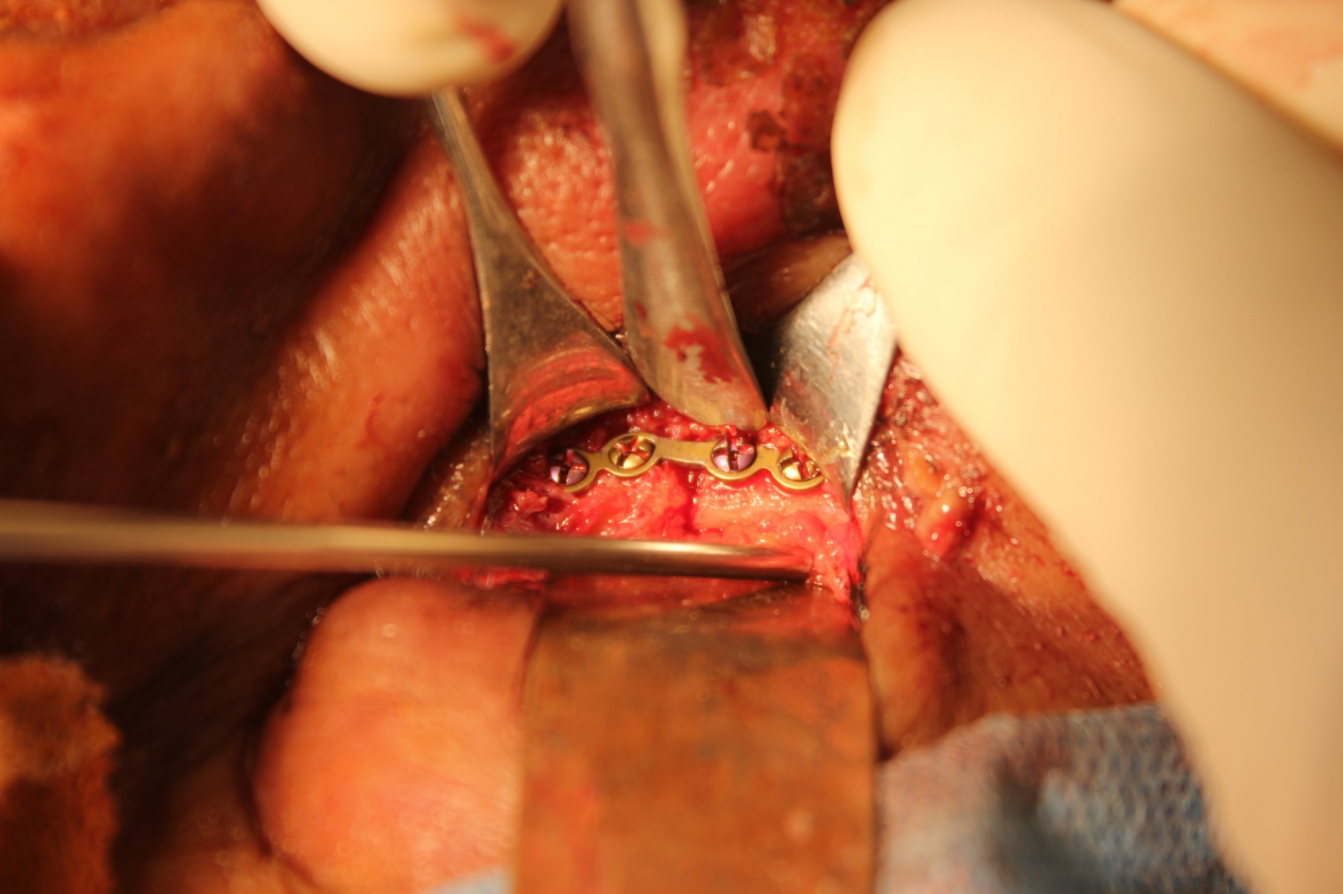
Miniplate fixation of the infraorbital rim

An open reduction and internal fixation of the right zygomaticomaxillary buttress and the right infraorbital rim was planned under general anesthesia. We placed an upper vestibular incision in the right side, and the fracture was exposed using subperiosteal dissection (Figure 5). We exposed the right infraorbital rim using a retroseptal transconjunctival approach (Figure 6). The lower eyelid was retracted using a Desmarres retractor. A corneal shield was placed to protect the eyeball, preventing corneal abrasions or tearing (Figure 7). The inferior fornix was held with toothed tissue-holding forceps, and an incision was placed into the palpebral conjunctiva using a Colorado tip (Stryker CMF, Chicago, IL, USA) electrocautery between the lowermost point of the eyelid and inferior fornix (Figure 8). Tenotomy scissors were used to locate the inferior orbital rim, and dissection was done until the periorbita was reached (Figure 9). A sub-periorbital dissection was done to expose the inferior orbital rim. The fracture was reduced using Rowe’s zygomatic elevator. After reduction, the zygomaticomaxillary buttress was fixed using a 2-mm Titanium L miniplate and five screws (2 mm x 6 mm; Stryker CMF, Chicago, IL, USA; Figure 10). The site was closed with 3-0 Vicryl sutures. The right infraorbital rim was fixed using a 1.5-mm Titanium orbital plate and four screws (1.5 mm x 6 mm; Stryker CMF, Chicago, IL, USA; Figure 11). The conjunctiva was closed with 4-0 Vicryl buried sutures to prevent corneal injuries from the sutures. A Frost suture was placed involving the lower tarsal plate and suspended from the right forehead region and retained for three days postoperatively. The patient was prescribed postoperative topical antibiotic drops (Ciprofloxacin) and eye lubricants (Carboxymethylcellulose) for five days. A Frost suture was placed in right lower eyelid for three days, suspending the lower eyelid.

## Discussion

The face plays an important role in the overall cosmesis of a person. Therefore, it is imperative to restore the face following trauma or ablative surgery to its original form and function. The zygomatic bone being convex and prominent in the face is more prone to get involved in maxillofacial fractures than other bones. The zygoma is a tetrapod structure with the following articulations [4]:

·      Maxilla at the zygomaticomaxillary buttress and infraorbital rim

·      The frontal bone at the frontozygomatic suture

·      Temporal bone forming the zygomatic arch

·      The sphenoid bone at the zygomaticosphenoid suture

Accurate three-dimensional reduction of the zygomaticomaxillary complex is of prime importance in establishing mid-facial width and contour of the lateral and inferior orbital borders.

The various signs and symptoms of zygomaticomaxillary complex fractures include swelling, pain, and tenderness in the cheek region; flattening of the cheek; subconjunctival ecchymosis; diplopia; enophthalmos; ecchymosis intraorally at the zygomaticomaxillary buttress; step deformity at the infraorbital rim; frontozygomatic suture, upper buccal sulcus, and zygomatic arch; and paresthesia over the infraorbital region.

**Figure 12:**
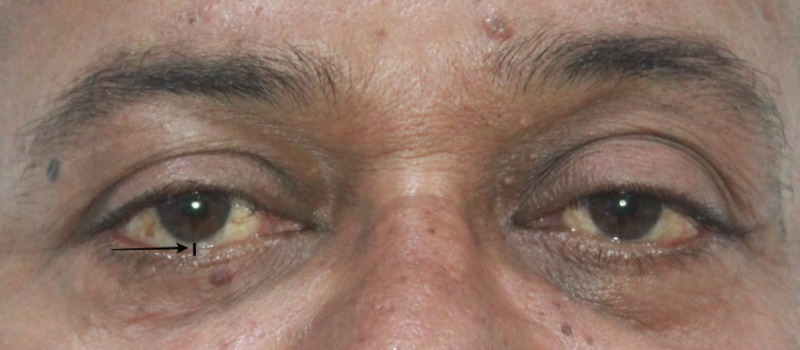
Postoperative evidence of a 2-mm scleral show in the right eye

**Figure 13:**
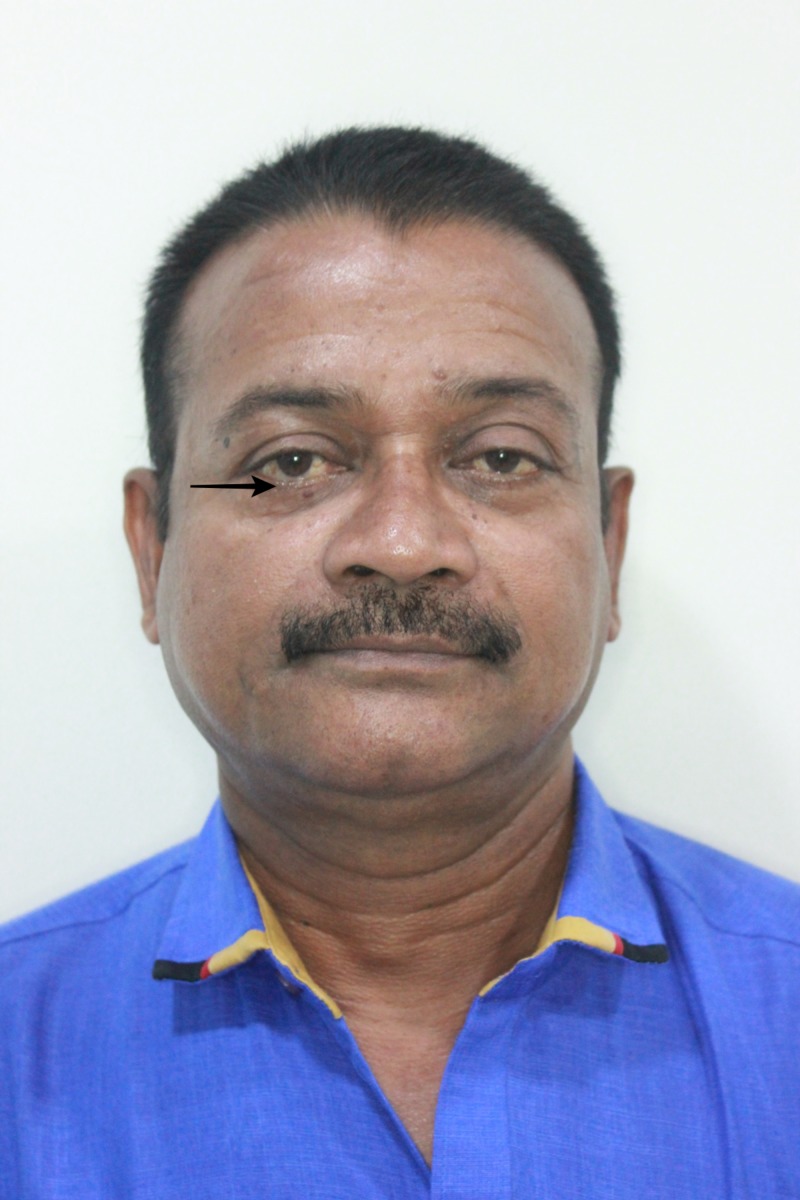
Extraoral frontal clinical picture demonstrating scarless repair of the right infraorbital rim

**Figure 14:**
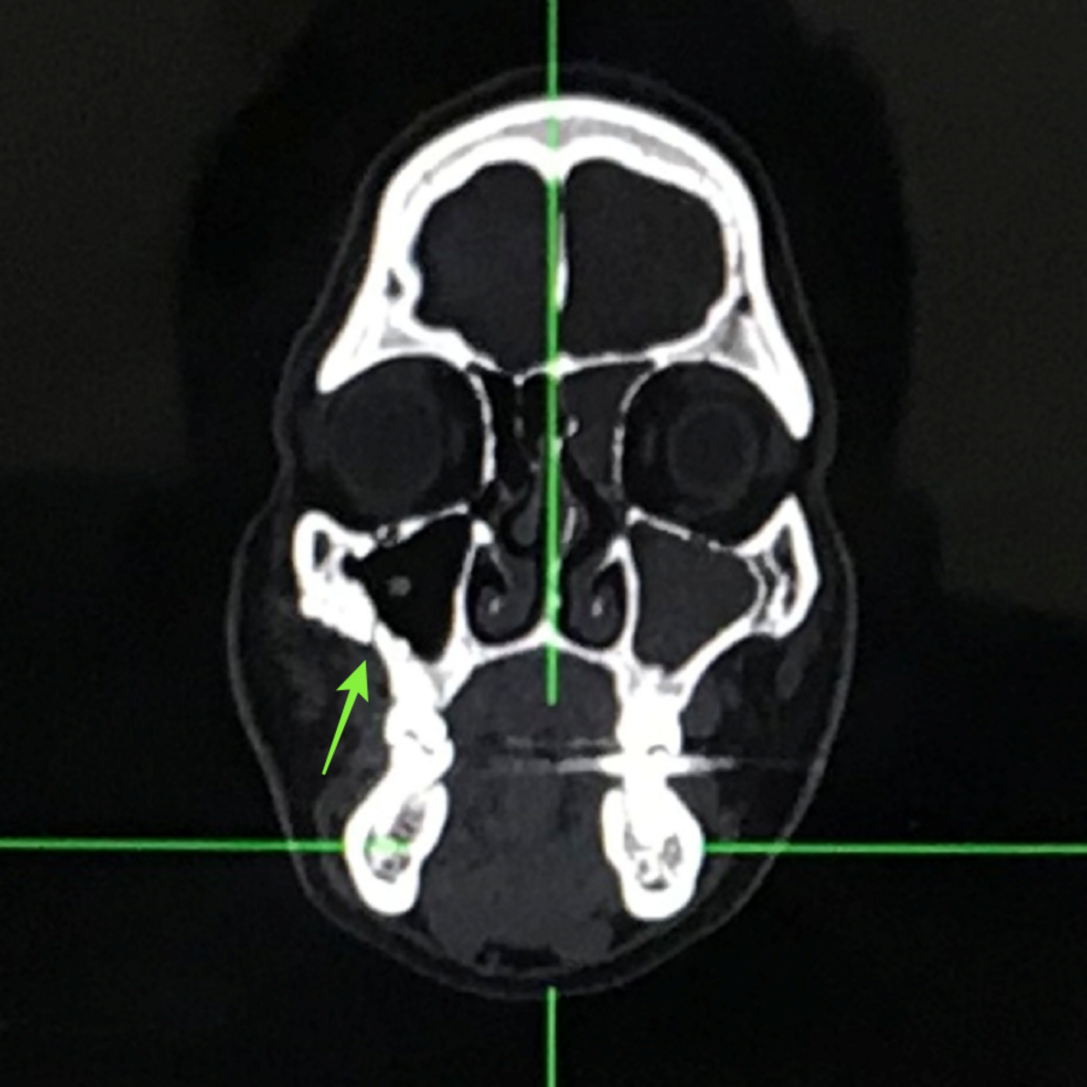
Postoperative computed tomography (coronal view) demonstrating acceptable fixation and lack of infection

**Figure 15:**
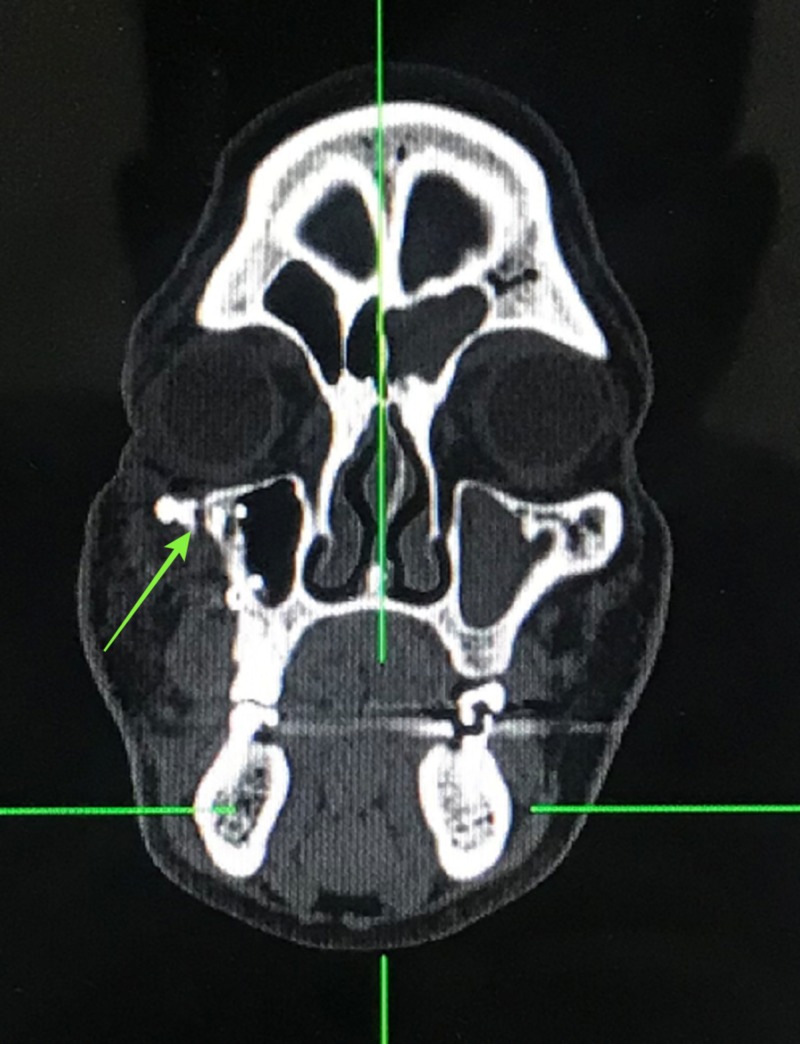
Postoperative computed tomography (coronal view) demonstrating acceptable fixation and lack of infection

In our case, the patient presented with a swelling in the right zygoma region with tenderness and step deformity in the right infraorbital rim and zygomaticomaxillary buttress intraorally. He had no diplopia or enophthalmos, and there were no restrictions in movement of the eyeball in all gazes. A detailed ophthalmic investigation revealed normal visual acuity. He had no associated trismus. A CT scan revealed a fracture at the zygomaticomaxillary buttress region and infraorbital rim (Figures 3-4). Based on the clinical and radiological findings, we planned for an open reduction and internal fixation of the zygomaticomaxillary buttress using an intraoral vestibular approach and fixation of the infraorbital rim using a retroseptal transconjunctival approach. Several transcutaneous approaches to access the infraorbital rim and orbital floor appear in the literature, namely, subciliary, subtarsal, and infraorbital approaches. According to Werther, the infraorbital incision is preferred for cases with marked edema which precludes the accurate placement of subciliary or subtarsal incisions [5]. However, higher rates of scleral show and ectropion have been reported with a subciliary incision than with a transconjunctival approach according to Patel et al. and Appling et al. [6-7]. In terms of speed and easy access, the subtarsal incision has its advantages, although noticeable scarring and persistent edema have been documented as a result of the subtarsal approach [8-9]. We used a retroseptal transconjunctival approach to access the infraorbital rim. In this approach, the conjunctiva was dissected from behind the orbital septum down to the bony orbit. In the transconjunctival preseptal approach, the orbital septum is incised below the tarsus and followed down to the orbital rim. The lateral canthotomy along with a transconjunctival incision allows the periosteum to be elevated superiorly to repair the frontozygomatic suture. However, we required the exposure of infraorbital rim alone, and, thus, lateral canthotomy was not performed. According to Holtmann et al., the average skin-to-fracture-exposing time was five to eight minutes for the infraorbital rim and subtarsal incision [10]. The subciliary incision was developed in 15 minutes [10]. According to Santosh and Giraddi, the average time to expose a fracture site using a preseptal transconjunctival approach was 21 minutes [11]. In our case, we took nine minutes to expose the fracture site at the infraorbital rim via a retroseptal transconjunctival approach. In addition, we believe this approach offers a distinct advantage of not violating the orbital septum, thus reducing the chances of lower eyelid malposition. The amount of exposure was enough to plate the infraorbital rim (Figure 11). However, a lateral canthotomy would provide access to the lateral orbital margin including the frontozygomatic suture if fixation was required at these regions. The biggest advantage of the retroseptal transconjunctival approach is that it offers scar-free, direct access to the infraorbital rim and orbital floor. The patient was evaluated six months after the surgical operation, and we found no evidence of scar, ectropion, entropion or lower eyelid retraction (Figure 12). A CT scan at the six-month postoperative follow-up evaluation revealed acceptable reduction and fixation of the fracture segments. We found no signs of infection at the fixation sites (Figures 13-14). However, the patient exhibited 2 mm of scleral show, which we attribute to the removal of the Frost suture on the third postoperative day (Figure 15). We recommend retaining the Frost suture for seven to 10 days, thus minimizing the chances of lower eyelid malposition.

## Conclusions

The transconjunctival retroseptal approach proves to be an effective and esthetic surgical approach to access the infraorbital rim and orbital floor. We recommend acquiring a detailed knowledge of the applied orbital anatomy. Proper training and meticulous surgical handling are imperative in executing a transconjunctival retroseptal technique, even for a young surgeon. The transconjunctival retroseptal technique along with a lateral canthotomy could be the mainstay approach in the future for accessing the orbital rim or orbital floor, including the lateral orbital wall inclusive of the frontozygomatic suture.
